# Post-Golgi Trafficking and Transport of Cell Wall Components

**DOI:** 10.3389/fpls.2018.01784

**Published:** 2018-12-07

**Authors:** Rosalie Sinclair, Michel Ruiz Rosquete, Georgia Drakakaki

**Affiliations:** Department of Plant Sciences, University of California, Davis, Davis, CA, United States

**Keywords:** post-Golgi trafficking, *trans*-Golgi Network, endosome, polysaccharide trafficking, cell wall, endomembrane trafficking, glycome analysis, SNARE

## Abstract

The cell wall, a complex macromolecular composite structure surrounding and protecting plant cells, is essential for development, signal transduction, and disease resistance. This structure is also integral to cell expansion, as its tensile resistance is the primary balancing mechanism against internal turgor pressure. Throughout these processes, the biosynthesis, transport, deposition, and assembly of cell wall polymers are tightly regulated. The plant endomembrane system facilitates transport of polysaccharides, polysaccharide biosynthetic and modifying enzymes and glycoproteins through vesicle trafficking pathways. Although a number of enzymes involved in cell wall biosynthesis have been identified, comparatively little is known about the transport of cell wall polysaccharides and glycoproteins by the endomembrane system. This review summarizes our current understanding of trafficking of cell wall components during cell growth and cell division. Emerging technologies, such as vesicle glycomics, are also discussed as promising avenues to gain insights into the trafficking of structural polysaccharides to the apoplast.

## The Plant Cell Wall

Consisting of a complex weaving of macromolecules, the cell wall is essential for many cellular processes such as development, cell integrity, signal transduction, defense, and maintenance of turgor pressure (Collins et al., 2003; Cosgrove, 2005, 2016). Roughly 40 cell types make up a plant with their cell walls determining their unique shape and function (Somerville et al., 2004; De Souza et al., 2015; Cosgrove, 2016; Chebli and Geitmann, 2017). The structurally dynamic and heterogeneous primary walls of young plant cells are predominantly comprised of cellulose microfibrils embedded in a matrix of pectin, hemicellulose, and glycoproteins (McCann et al., 1992; Somerville et al., 2004; Burton et al., 2010). Although during the last 20 years many cell wall biosynthetic enzymes have been identified, the understanding of the mechanisms facilitating polysaccharide transport is far from comprehensive. With a better understanding of cell wall component trafficking pathways, detailed models of cell wall deposition and maturation can be constructed, providing insights into the dynamic organization of cell wall during plant growth and in response to environmental cues. This review focuses on transport and deposition of cell wall components during primary cell wall formation. For a comprehensive review of secondary cell wall biosynthesis see (Meents et al., 2018).

Xyloglucan (XyG) represents the predominant hemicellulose in the primary cell walls of eudicots and non-graminaceous monocots. XyG is a β-1,4 glucan featuring a regular pattern of substitutions occurring along the xylose residue on the glucan backbone. Xylose substitutions consist of galactose, O-acetylated galactose and fucose residues (Keegstra, 2010; Scheller and Ulvskov, 2010; Pauly and Keegstra, 2016; Kim et al., 2018). Pectin, a complex heterogeneous assembly of polysaccharides whose structural backbones contain galacturonic acid residues, constitutes a major part of the matrix into which cellulose microfibrils are embedded (Atmodjo et al., 2013). Both pectin and hemicellulose are synthesized by Golgi localized enzymes and thus require transport to the plasma membrane (PM), a critical yet poorly understood role of the endomembrane system. In contrast, cellulose, another key component of cell walls, is synthesized at the PM by Cellulose Synthase Complexes (CSCs) (McFarlane et al., 2014). Although the transport of pectin, hemicellulose and CSCs to the PM is currently thought to utilize the conventional ER–Golgi–*trans*-Golgi Network (TGN)–PM traffic route, unconventional pathways have been postulated. This review primarily focuses on TGN dependent pathways. For recent reviews on unconventional protein secretion (UPS) see (Davis et al., 2016; van de Meene et al., 2017; Wang X. et al., 2017).

## Multiple Factors May Coordinate Tgn-Mediated Transport of Cell Wall Components

Accurate spatial and temporal delivery of cell wall material is essential in choreographing cellular responses to the environment such as those elicited by pathogens. The endomembrane system’s intricate array of molecular players orchestrates the timely delivery of cargos crucial to cell functions, and cell wall components are no exception. The TGN is the membrane compartment on the *trans*-side of Golgi responsible for sorting and packaging cargo molecules targeted to the PM or vacuoles (Roth et al., 1985; Griffiths and Simons, 1986; Kang et al., 2011; Rosquete et al., 2018). Unlike in other eukaryotes, plant TGN also serves as an early endosome (Dettmer et al., 2006; Viotti et al., 2010). A specialized role of the Golgi apparatus and the TGN in plants is the biosynthesis and sorting of cell wall components including biosynthetic enzymes, structural proteins and the matrix polysaccharides hemicellulose and pectin (Cosgrove, 2005; Worden et al., 2012; Kim and Brandizzi, 2016; van de Meene et al., 2017).

The function of the TGN is regulated by a plethora of factors including RAB GTPases, soluble *N*-ethylmaleimide-sensitive factor attachment protein receptors (SNAREs), tethers, accessory proteins, as well as vesicle pH and lipid composition (Ebine and Ueda, 2015; Zhen and Stenmark, 2015; Rosquete et al., 2018) SNARE proteins mediate membrane fusion (Ueda et al., 2012; Bombardier and Munson, 2015), with syntaxins representing a sub-family of SNAREs. Arabidopsis contains distinct syntaxins of plants (SYPs) localized to different compartments of the endomembrane system (Sanderfoot et al., 2000). SYP61, a plant SNARE interacting with SYP42/3 and VTI12 members in the TGN, appears central to the organelle’s trafficking functions (Drakakaki et al., 2012; Hofmann, 2017; Li et al., 2017), discussed in depth in this review.

**FIGURE 1:**
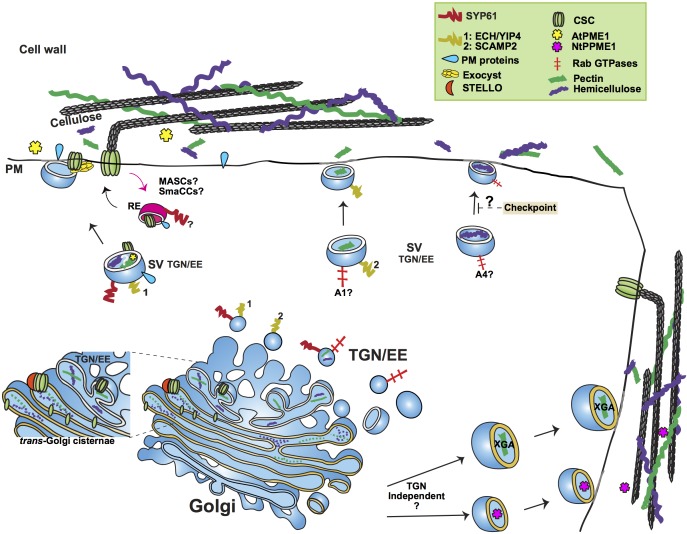
Simplified outline of post-Golgi transport pathways involved in trafficking of cell wall components. The plant *trans*-Golgi Network/early endosome (TGN/EE) mediates the post-Golgi trafficking of the cell wall structural polysaccharides pectin and hemicellulose and cell wall biosynthetic and modifying enzymes, such as Cellulose Synthase Complexes (CSC) and Pectin Methylesterase1. Pectin and hemicellulose are synthesized in Golgi, from where they are transported to the plasma membrane (PM) in secretory vesicles (SV) whose identity is under study. An important question remains whether pectin and hemicellulose are transported in the same type of TGN vesicles. Genetic evidence points toward a role of the SYP61/ECH/YIP4 compartment in the secretion of pectin and hemicellulose. SCAMP2 vesicles have also been shown to transport pectin. Based on the analysis of mutants’ cell wall composition, a functional specialization of RABA GTPases has been suggested, with RABA1 and RABA4 specifically regulating the transport of pectin and hemicellulose, respectively. A hemicellulose structural checkpoint that feeds back to post-Golgi secretory traffic has been proposed. Not only TGN dependent but also TGN independent routes have been shown to mediate the trafficking of cell wall polysaccharides. Two examples of the latter are the secretion of the specialized Xylogalacturonan (XGA) in border root cells of alfalfa and the secretion of Pectin Methylesterase1 in tobacco cells (NtPPME1). CSCs are assembled in Golgi, aided by STELLO proteins. Various compartments (MASCs/SmaCCs and SYP61) have been implied in both the biosynthetic and recycling traffic of CSCs. The exocyst complex is provided as an example of a tethering complex with a crucial function in post-Golgi trafficking by facilitating the secretion of CSCs. The SYP61 compartment is involved in the transport of not only CSCs but also cell wall modifying enzymes, as indicated by the presence of Arabidopsis Pectin Methylesterase1 (AtPME1) in the SYP61 vesicle proteome. RE, Recycling Endosome; A1, RABA1; A4, RABA4.

RAB GTPases have been shown to confer specificity to vesicle traffic and mediate membrane fusion between a donor and an acceptor compartment (Woollard and Moore, 2008; Ebine et al., 2011; Lunn et al., 2013b; Bhuin and Roy, 2014). There are 57 Arabidopsis *RAB* genes, split into 8 clades and further classified into subclades of various sizes (Zhang et al., 2007). Vernoud et al. (2013) in trafficking of cell wall components. In a study that used Fourier transformed infrared spectroscopy to evaluate cell wall composition, the percentage of pectin, cellulose and hemicellulose within the cell wall was affected by single mutants of RABA1, RABA2, and RABA4, respectively (Lunn et al., 2013a). Based on this, the authors hypothesized specialized roles for those GTPases in the transport of specific types of polysaccharides from Golgi to the cell surface (Lunn et al., 2013a). Interestingly, RABA2, together with RABA3, has also been implied in trafficking of material to forming cell plates during cytokinesis (Chow et al., 2008) opening the question whether these two RABs are involved in the trafficking and deposition of polysaccharides at the cell plate. Though exciting, further investigation is needed into the possible specificity of these RAB GTPases (see Figure 1).

Although the roles of TGN resident tethers, adaptor proteins and vesicle lipid composition in post-Golgi protein trafficking and TGN functional compartmentalization have started to emerge, very little or nothing is known on how they regulate/impact TGN-mediated polysaccharide trafficking (Kim and Bassham, 2011; Bashline et al., 2013; Di Rubbo et al., 2013; Vukasinovic and Zarsky, 2016; Wattelet-Boyer et al., 2016; Ravikumar et al., 2017; Boutte, 2018).

## Trafficking of Cell Wall Structural Proteins

Structural cell wall proteins such as arabinogalactan proteins (AGPs), extensins and proline-rich proteins (PRPs) play a role in defining the cell wall’s physical functional properties (Showalter and Basu, 2016a; Johnson et al., 2017a,b). However, we know very little about their location within the cell wall, their specific roles and even less about their intracellular trafficking routes. Regarding the latter, both Golgi-dependent and Golgi-independent pathways have been suggested to contribute to the secretion of cell wall proteins (Ellis et al., 2010; Tan et al., 2012; Poulsen et al., 2015; Showalter and Basu, 2016a,b).

Glycosylation is a common post-translational modification amongst PM and cell wall proteins (Nguema-Ona et al., 2014). Interestingly, a recent work combining high-resolution tandem mass spectrometry and available subcellular localization data, established links between the most prominent N-glycan structures present in a given glycoprotein and the protein’s distribution in the endomembrane system, in turn reflecting the glycoprotein’s trafficking pattern. The presence of complex N-glycans correlated with Golgi/PM localization while paucimannose structures were associated with extracellular glycoproteins (Zeng et al., 2018).

Arabinogalactan proteins are thought to be secreted through a Golgi-dependent pathway, supported by proteomic analysis of Golgi-enriched fractions (Ford et al., 2016). Contrary to this model, a study using tobacco cells detected transiently expressed AGPs glycosyltransferases in a double-membraned, Exocyst-Positive Organelle (EXPO) (Poulsen et al., 2014). EXPO has been implicated in Golgi-independent, unconventional secretion of, mostly, proteins that do not carry a signal peptide (leaderless proteins) (Wang et al., 2010; Poulsen et al., 2014, 2015; Davis et al., 2016). These findings suggest that Golgi-independent pathways may also mediate secretion of AGPs. However, such a view requires further examination as both the glycosyltransferases and the AGP protein core are not leaderless and are thus expected to follow the canonical secretory pathway.

## Trafficking of Cell Wall Biosynthetic and Modifying Enzymes

The Cellulose Synthase Complexes assemble in the Golgi apparatus and are then transported to the PM, both events likely being assisted by STELLO proteins (McFarlane et al., 2014; Lampugnani et al., 2018). Tracking of CSCs at the PM has shown them moving linearly along cortical microtubules (MT) supporting the MT-cellulose alignment hypothesis (Paredez et al., 2006).

Perhaps the most studied cell wall-related trafficking process, the transport of CESAs has been proposed to be mediated by the plant specific endomembrane compartments SmaCC (small CESA compartment) and MASCs (microtubule-associated cellulose synthase compartments) (Crowell et al., 2009; Gutierrez et al., 2009). The role of MASCs/SmaCCs are involved in secretory and endocytic/ recycling routes of CESAs (Crowell et al., 2009; Gutierrez et al., 2009). Recently, the plant-specific protein PATROL1 (PTL1) and members of the exocyst complex have been associated with vesicle docking and secretion of CSCs during primary cell wall formation (Zhu et al., 2018).

Clathrin-mediated endocytosis and recycling rates influence the steady-state levels of CSCs at the PM (Bashline et al., 2013; Lei et al., 2015; Sanchez-Rodriguez et al., 2018). The plant-specific T-PLATE complex, a major adaptor module for clathrin-mediated endocytosis, was recently shown to recognize CSCs for their internalization (Gadeyne et al., 2014; Sanchez-Rodriguez et al., 2018).

CESAs are cargo of the SYP61 vesicles, implicating the SYP61 TGN compartment in the post-Golgi trafficking of these enzymes (Gutierrez et al., 2009; Drakakaki et al., 2012). This is supported by the finding that CESTRIN, a small molecule that reduces the motility of CSCs at the PM, increases the association of CESAs with SYP61 vesicles (Worden et al., 2015). However, it remains unclear whether the CESAs found in the SYP61 compartment represent endocytic or secretory pools, or both. Further, CESAs secretion is affected in mutants of VHA-a1, a TGN specific proton pump isoform that partially localizes to the SYP61 compartment where it plays a prominent role in the establishment of the vesicle luminal pH (Luo et al., 2015).

Similarly to CSCs, callose biosynthesis enzymes such as Glucan Synthase-Like (GSLs) are trafficked to the PM prior to the initiation of polysaccharide synthesis (Brownfield et al., 2007, 2008; Toller et al., 2008). Not much is known about the trafficking routes of the twelve Arabidopsis GSL isoforms although several of them were identified in the proteome of SYP61 TGN/EE vesicles (Drakakaki et al., 2012). The latter suggests a canonical secretory route to the PM, with the likely involvement of the EXOCYST tethering complex in the final event of fusion to the PM, as indicated by callose deposition studies in Arabidopsis trichomes (Kulich et al., 2018). Interestingly, trafficking of the GSL isoform PMR4 to sites of callose accumulation during the plant response to the pathogen *Blumeria graminis* f. sp. *hordei* has been suggested to occur via unconventional pathways, with the involvement of either multivesicular bodies (Bohlenius et al., 2010) or exosomes (Ellinger et al., 2013). Despite these observations, the trafficking of GSLs during different growth stages and stress conditions needs further investigation.

Cell wall associated enzymes such as apoplastic glycosidases contribute to the modification of polysaccharides during cell wall assembly creating cell wall structural diversity (Showalter, 1993; De Caroli et al., 2011; Gunl et al., 2011a,b; Sampedro et al., 2012; Frankova and Fry, 2013; Pauly and Keegstra, 2016). These proteins are thought to traffic through the secretory pathway; however, recent evidence indicates that multiple pathways may be involved. The main known cell wall modifying enzyme acting on XyG, β-GALACTOSIDASE 10 (AtBGAL10), has three distinct N-glycosites, two with multiple high-mannose structures and the third containing paucimannose structures (Sampedro et al., 2012). Based on the presence of high-mannose structures, Zeng et al. (2018) speculated that an UPS pathway could be involved in the trafficking of β-GALACTOSIDASE 10.

Pectin methylesterase1 (AtPME1), a pectin modifying enzyme, was identified in the proteome of Golgi and also in those of SYP61 and VHA-a1 TGN vesicles, indicating that a Golgi-TGN route is involved in its trafficking to the PM (Drakakaki et al., 2012; Nikolovski et al., 2012; Groen et al., 2014; Heard et al., 2015). However, a different, Golgi-PM pathway that bypasses TGN was identified for the trafficking of tobacco’s pollen-specific NtPPME1 (Wang H. et al., 2016).

## Transport of Structural Polysaccharides

Compared to the number of studies that have provided insights into the secretion of cell wall biosynthetic proteins, less is known on the secretion of structural polysaccharides. Our current knowledge of cell wall polysaccharide transport results primarily from immunoelectron microscopy studies (EM) (Moore et al., 1986, 1991; Moore and Staehelin, 1988; Lynch and Staehelin, 1992; Young et al., 2008; Kang et al., 2011). However, limitations arise from the incompatibility of staining with traditional antibodies, and electron microscopy itself, with live imaging, restricting the experiments to sections of embedded tissue. Despite such limitations, a few studies have shed light into the intracellular distribution of plant polysaccharides.

A seminal study in sycamore maple (*Acer pseudoplatanus*) cells detected the XyG backbone in *trans*-Golgi cisternae, whereas fucosylated XyG side chains were identified in both the *trans* cisternae and the TGN (Zhang and Staehelin, 1992). This suggests a developmental “assembly line” consisting of the initial biosynthesis of the backbone followed by the addition of side chains in Golgi sub-compartments, with the TGN transporting, mostly, fully substituted XyG (Zhang and Staehelin, 1992). In the same study, low-methylesterified pectin backbone, detected by the antibody JIM5, was found distributed in the *cis*- and *medial*-Golgi and at the cell wall whereas high-methylesterified pectin, detected by JIM7, was localized to the *medial*- and *trans*-Golgi, in secretory vesicles and at the cell wall. These observations indicate that pectins undergo maturation while they are delivered to the *trans*-Golgi, and that high-methylesterified pectin is the predominantly secreted form (Zhang and Staehelin, 1992). They also suggest that conventional post-Golgi trafficking pathways are used by both XyG and pectin. Importantly, the colocalization of XyG and pectin epitopes in transport vesicles of clover root tips indicate that TGN vesicles can potentially carry both polymers (Lynch and Staehelin, 1992).

Recent studies point to a role of cortical MTs in pectin deposition at the cell wall. Mucilage secretion was shown to be targeted to PM domains lined by abundant cortical MTs. Corroborating this observation, the temperature-sensitive MT mutant *mor1-1* exhibited a decreased mucilage secretion in seeds, in the same study (McFarlane et al., 2008). In addition, the fragile Fiber1 (FRA1) kinesin has been implicated in pectin deposition (Kong Z. et al., 2015; Zhu et al., 2015).

Not surprisingly, the cell type has been shown to determine the trafficking fate of vesicles transporting cell wall polysaccharides. Xylogalacturonan (XGA), a pectin variant secreted by root border cells, is transported by distinct large vesicles, released from the *trans*-Golgi cisternae. XGA-loaded vesicles were shown to fuse with the PM in alfalfa border root cells but not in peripheral cells indicating the existence of regulatory mechanisms conferring cell type specificity to the trafficking and secretion of specialized polysaccharides (Wang P. et al., 2017; see Figure 1).

As aforementioned, proteomic analysis of SYP61 vesicles identified proteins involved in cell wall development (Drakakaki et al., 2012). These include the TGN-resident complex formed by ECHIDNA (ECH) and the YPT/RAB GTPase interacting Proteins 4a and 4b (YIP4a and YIP4b), implicated in the secretion of pectin and XyG (Gendre et al., 2011, 2013). Inhibition of cell elongation and altered secretion of XyG and RGI pectins were shown in mutants of YIP4A, YIP4B, and ECH (Gendre et al., 2011, 2013). Further, antibodies for fucosylated XyG have been shown to label a RABA4b TGN compartment in Arabidopsis (Kang et al., 2011). Because RABA4b and SYP61 colocalize at TGN, this observation further supports a role for the SYP61 compartment in the trafficking of structural polysaccharides. The PM resident syntaxin SYNTAXIN OF PLANTS121 (SYP121) has been shown to form a SNARE complex with SYP61 mediating the secretion of PM protein cargo (Geelen et al., 2002; Hachez et al., 2014). A role for SYP121 in the secretion of SYP61 polysaccharide cargo is thus likely. Interestingly, the AtSYP121 homolog, NtSyr1, seems dispensable for polysaccharide transport in tobacco cells based on transient studies, an observation that awaits thorough testing in Arabidopsis and other plant systems (Leucci et al., 2007; see Figure 1).

Anti-pectin antibodies stain SCAMP2 (Secretory Carrier Membrane Protein 2) vesicles in tobacco BY-2 cells, suggesting the involvement of that vesicle population in the transport of pectins (Toyooka et al., 2009). The SCAMP protein structure is well conserved in eukaryotes (Law et al., 2012). In humans, SCAMP2 regulates exocytosis by forming a membrane fusion complex with the small GTPase Arf6 (ADP-ribosylation factor 6), phospholipase D1 (PLD1), and SYNTAXIN 1 (Liu et al., 2002, 2005). It is plausible that SCAMP2, via a similar mechanism, regulates exocytosis of pectin and other polysaccharide cargo in plant cells.

A role for the Exocyst complex in pectin deposition has been suggested, based on genetic evidence. Mutants of the Exocyst subunits SEC8 and EXO70A1 show reduced pectin accumulation in the seed coat. Further, reduced pectin deposition was also observed in a gain-of-function mutation of ROH1, an interactor of the exocyst subunit Exo70A1, supporting the involvement of this tethering factor in polysaccharide transport (Kulich et al., 2010).

Intriguingly, the structure of cell wall polysaccharides, rather than their levels, seems to influence their trafficking, as suggested by the formation of intracellular aggregates containing xyloglucan and deesterified homogalacturonan in mutants of the XyG biosynthetic enzyme galactosyltransferase MUR3 (Kong Y. et al., 2015). It is thus likely that XyG structure checkpoints that feedback to post-Golgi secretory traffic exist. Interestingly, a role for MUR3 in maintaining the organization of Golgi via its interaction with actin filaments has been suggested, hinting at a link between actin cytoskeleton and structural polysaccharides transport (Tamura et al., 2005).

## Polysaccharide Transport to the Cell Plate

During plant cytokinesis, a cell plate that partitions the cytoplasm of the dividing cell is formed *de novo* (Samuels et al., 1995; Staehelin and Hepler, 1996; Jurgens, 2005; Drakakaki, 2015). Such event requires the coordinated action of cytoskeletal transitions and endomembrane trafficking (Samuels et al., 1995; Otegui et al., 2001; Otegui and Staehelin, 2004; Segui-Simarro et al., 2004; Lee and Liu, 2013; Smertenko et al., 2017). Cell plate development occurs in four stages that exist simultaneously. It requires the directed and choreographed accumulation of post-Golgi vesicles to the phragmoplast at the division plane and removal/recycling of excess material (Samuels et al., 1995; Segui-Simarro et al., 2004; Drakakaki, 2015; Smertenko et al., 2017). The deposition of cell wall polymers transforms the lumen of this membrane compartment into a new cross wall, physically separating the daughter cells (Drakakaki, 2015; Smertenko et al., 2017). Whereas a number of studies have investigated membrane dynamics (van Oostende-Triplet et al., 2017), few reports exist on polysaccharide deposition and its explicit role during cell plate maturation as summarized in recent reviews (Drakakaki, 2015; Chen et al., 2018).

Vesicle trafficking during cell plate formation is controlled by many molecular players, including Rab GTPases, SNAREs, tethering factors and other regulatory proteins (reviewed in (McMichael and Bednarek, 2013; Boruc and Van Damme, 2015; Drakakaki, 2015; Smertenko et al., 2017). Two well-studied factors are RABA2A, which regulates the delivery of TGN derived vesicles to the leading edge of the cell plate (Chow et al., 2008) and the cytokinesis specific SNARE KNOLLE, which catalyzes homotypic fusion of vesicles at the cell plate (Lauber et al., 1997; Assaad et al., 2001; Heese et al., 2001; Zheng et al., 2002; Zhang et al., 2011; El Kasmi et al., 2013; Karnahl et al., 2018).

The delivery and deposition of cell wall materials to the cell plate has been primarily studied with electron and fluorescence microscopy utilizing polysaccharide-specific antibodies. The current notion is that structural polysaccharides such as hemicellulose and pectins are transported in trans-Golgi derived secretory vesicles to the expanding and maturing cell plate (Moore and Staehelin, 1988; Samuels et al., 1995; Toyooka et al., 2009; Drakakaki, 2015). The presence of XyG is detected at early stages with enrichment in later stages (Moore and Staehelin, 1988). In Arabidopsis, the pectin backbone has been detected at the cell plate in methylesterified form (Clausen et al., 2003; Rybak et al., 2014). In red clover root tips, RGI and polygalacturonic acid labeling were observed at the middle lamella, the mature central layer of the cell plate that serves as a glue between adjacent cells. However, RGI was not detected at the early cell plate, suggesting that acidic polysaccharides may be deposited at later stages of cross wall development (Moore and Staehelin, 1988). In addition to Golgi/TGN derived polysaccharides (Moore and Staehelin, 1988), internalized pectin glycans have been implicated in cell plate formation (Baluska and Volkmann, 2002; Dhonukshe et al., 2006), a notion that awaits further investigation.

Callose and cellulose are vital luminal polysaccharides of the cell plate as supported by genetic evidence (Zuo et al., 2000; Beeckman et al., 2002; Chen et al., 2009; Thiele et al., 2009; Guseman et al., 2010; Gu et al., 2016). The relative spatiotemporal distribution of callose, cellulose and their biosynthetic enzymes during the different stages of cell plate formation is not fully elucidated. According to current thinking, callose is transiently incorporated for mechanical support during the middle/late stages of cell plate formation and is ultimately replaced by cellulose, for a more rigid luminal network (Samuels et al., 1995; Thiele et al., 2009). However, recent live imaging of cellulose synthase has shown that it accumulates at the early tubulo-vesicular network stage, concomitant with cellulose (Miart et al., 2014). Cell wall biosynthetic enzymes in the Cellulose Synthase Like-D family (CslD) exhibit high homology to CESAs and are also involved in cell plate formation (Gu et al., 2016). The cell cycle-regulated CslD5 is localized at early cell plate stages where it presumably produces a cellulose-like molecule, as previously shown for CslD3 in polarized root hair growth (Park et al., 2011).

Stains with both the synthetic chemical dye β-glycosyl Yariv and the monoclonal antibody LM14 have shown that AGPs, together with polysaccharides, contribute to cell plate expansion (Yu and Zhao, 2012; Rybak et al., 2014). In addition, EXTENSIN3, a hydroxyl-proline-rich glycoprotein has been implicated in cytokinesis (Hall and Cannon, 2002; Cannon et al., 2008). It is hypothesized that the self-assembled EXTENSIN network can provide mechanical support during the expansion of the cell plate, presumably via interaction with pectins (Cannon et al., 2008).

Although several endomembrane proteins have been associated with cell plate assembly, including the aforementioned SCAMP2 and the exocyst complex, little is known on their direct involvement in polysaccharide transport to the cell plate (Toyooka et al., 2009; Rybak et al., 2014).

## Emerging Technologies to Dissect Polysaccharide Transport

Live imaging of polysaccharides remains technically very challenging, making it difficult to assess the colocalization of a particular polysaccharide cargo with subcellular protein markers for specific vesicle populations. However, thanks to recent light microscopy advances that allow the use of photoactivatable and photoconvertible forms of cell wall associated proteins, combined with improved resolution imaging of carbohydrates, our knowledge of cell wall components trafficking and deposition is expected to quickly expand (Fernandez-Suarez and Ting, 2008; Toyooka and Kang, 2014; Mishin et al., 2015; Wang B. et al., 2016; Komis et al., 2018; Voiniciuc et al., 2018). In addition, field emission scanning electron microscopy (FESEM) with nanogold affinity tags affords resolution of spatial location and conformation of cell wall polymers and has proved useful to study XyG-cellulose interactions at the cell wall although this approach does not allow for live imaging (Zheng et al., 2018).

Live imaging of cell wall glycans using small oligosaccharide probes modified via click chemistry, together with polysaccharide dyes, can also contribute useful insights (Mravec et al., 2014). However, the toxicity of click chemistry reagents limits their use in live imaging experiments (Anderson et al., 2010, 2012; Wallace et al., 2012; Wang B. et al., 2016). The ever-expanding palette of metabolically labeled glycans could become a great asset for the dissection of cell wall metabolism once adapted for live imaging (Hoogenboom et al., 2016; Zhu and Chen, 2017). Cell wall glycan-directed antibodies are an elegant alternative for the identification of plant cell carbohydrates, and can be arrayed on automated large scale enzyme-linked immunosorbent assay (ELISA) platforms (glycome profiling) (Moller et al., 2008; Pattathil et al., 2010, 2012; Ruprecht et al., 2017). Cell-permeable, live imaging-compatible nanobodies represent another promising tool (Herce et al., 2017). Exemplifying their potential, a very recent study using a nanobody–epitope interaction-based protein labeling and tracking approach helped dissect a TGN/EE-to-*cis*-Golgi recycling pathway for vacuolar sorting receptors in *Nicotiana tabacum* cells (Fruholz et al., 2018).

To date, there are no suitable glycomic approaches that capture both the polysaccharide contents of specific vesicle populations and the detailed polysaccharide structures. By combining a vesicle isolation methodology, such as that established for the SYP61 compartment (Drakakaki et al., 2012), with vesicle glycome profiling, the roles of different vesicle populations in polysaccharide transport, in relation with developmental stages and responses to environmental stimuli, can be defined. In addition, glycomes of isolated vesicles, as described above, can be coupled with their respective proteomes, obtained with advanced mass spectrometry analysis (Parsons and Lilley, 2018), and with vesicle lipid composition profiling (Haraszti et al., 2016; Wattelet-Boyer et al., 2016; Boutte, 2018) for a better understanding of how the endomembrane system regulates cell wall transport and deposition.

Oligosaccharide mass profiling (OLIMP) utilizes specific glycosyl-hydrolases to digest cell wall polysaccharides to soluble oligosaccharides detectable by MALDI-TOF mass spectrometry (Obel et al., 2009; Gunl et al., 2010, 2011a) Analyzing Arabidopsis Golgi-enriched microsomal fractions by OLIMP showed that in the Golgi apparatus XyG oligosaccharides (XyGOs) with a lower level of xylose residue substitution by galactose and fucose are more abundant than XyGOs with a higher degree of substitution (Obel et al., 2009; Gunl et al., 2010, 2011a). However, overall this approach has limitations since it is not possible to separate contributions of the Golgi from the TGN or from endoplasmic reticulum contamination. The adequacy of OLIMP to characterize the polysaccharide cargo of specific vesicle populations has yet to be demonstrated.

Proximity tagging methods for protein localization at subcompartmental resolution, such as APEX, BioID, and SPPLAT, have the potential of not only solving the components of large protein complexes involved in cell wall biosynthesis and deposition but also their spatial distribution in membrane microdomains of subcellular compartments (Parsons and Lilley, 2018).

Further, a number of methodologies that have proved useful to study post-Golgi trafficking in other eukaryotes could be successfully implemented in the Plant field. One such method assessed the effect of ectopic intracellular localization of tethering factors on the trafficking fate of cognate vesicles (Wong and Munro, 2014). A set of Golgi-localized Golgin tethers was artificially targeted to mitochondria of mammalian cells, after which their ability to redirect Golgi-bound carriers to the ectopic destination was monitored. By adapting this and other methodologies, the role and specificity of putative polysaccharide trafficking regulators can be investigated.

All these approaches offer the potential to deepen our spatiotemporal understanding and help model the highly choreographed trafficking events leading to cell wall deposition during both normal and stressful growth conditions.

## Author Contributions

GD, MR, and RS designed and wrote the manuscript.

## Conflict of Interest Statement

The authors declare that the research was conducted in the absence of any commercial or financial relationships that could be construed as a potential conflict of interest.
